# Models of education for care workers in Australian nursing homes: improving the care of older people

**DOI:** 10.3389/fpubh.2025.1584889

**Published:** 2025-05-19

**Authors:** Jo-Anne Rayner, Anne-Marie Mahoney, Deirdre Fetherstonhaugh, Sandra Cowen

**Affiliations:** Australian Centre for Evidence Based Aged Care, La Trobe University, Bundoora, VIC, Australia

**Keywords:** aged care workforce, workforce training, multidisciplinary education, nursing home, online education

## Abstract

**Introduction:**

Providing high-quality care in nursing homes requires a skilled and knowledgeable workforce. The Australian nursing home workforce is made up predominantly of personal care workers who are unprepared educationally to provide care to older people with complex care needs. We describe the design, development, and implementation of two education packages for Australian personal care workers to improve their knowledge and confidence in providing optimal care to older people.

**Methods—pedagogy and education development:**

The first package was developed following original research, which explored the need for education focusing on recognising and reporting resident deterioration, the preferred way of delivering this education, and barriers to education. Time and costs were identified as barriers, indicating that short, modular education for care workers was required. Using a train-the-trainer model, this education (10 h of delivery) comprises eight modules designed to be delivered individually or over two days by a registered nurse. The second education package was commissioned work following the interim findings from the Australian Royal Commission into Aged Care Quality and Safety and focused on three identified areas of need: dementia care, a palliative approach to care, and oral and dental care. Also modular, the second package has a learner-centric approach for the multidisciplinary aged care team and is freely available online.

**Results:**

User acceptability testing found the first package to be of high quality, easy to deliver, and the content can be adapted to meet individuals’ different learning styles, knowledge needs, and time availability. User acceptability testing of the second education package was undertaken in the development phase. This package has an international reach and continues to provide a popular single, easily accessible site for personal care worker education, who comprise 60% of users.

**Conclusion:**

While research suggests that personal care workers prefer face-to-face interactive education and training, the COVID-19 pandemic has changed the education landscape, and care workers are also embracing online learning. These education packages meet the needs of the personal care workforce, providing choice and flexibility: interactive learning in the context of care delivery or freely available online learning that can be undertaken in personal time.

## Introduction

The number of older Australians living permanently in nursing homes^1^ increased by 8.3% between 2012 and 2022, from 167,000 to 181,000 people (1). Most older people living permanently in nursing homes are women aged 85 years or older who have high care needs relating to changed cognition and behaviors (68%), assistance with activities of daily living (68%), and complex health care (58%) (1). The Australian Commonwealth government is responsible for aged care legislation, policy, funding, and regulation. Approved nursing home service providers are funded per place (beds) and regulated by legislated minimum care standards. Australian nursing home service providers include for-profit (private businesses), not-for-profit (religious, charitable, and community organisations), and the public sector (state and local governments) (2).

Providing high-quality and safe care in nursing homes requires an educated and skilled workforce. There is considerable diversity in Australian nursing home workforce arrangements, as historically the Commonwealth government did not mandate minimum staffing numbers or skills mix (3). However, in response to findings from the Australian Royal Commission into Aged Care Quality and Safety (4) in July 2023 it was mandated, that all approved nursing home providers must have at least one registered nurse (RN) on site, on duty 24 h a day, seven days a week (5).^2^ Generally, the nursing home workforce consists of RNs and enrolled nurses (ENs), and personal care workers (PCWs), variously called personal support workers, personal care attendants, caregivers, or assistants in nursing (6).^3^ Australian nurses have tertiary education and are registered with the Australian Health Practitioner Regulation Agency, with defined scopes of practice (7, 8). PCWs, who are unregistered, have no defined or legal scope of practice and variable education and training, comprise 71.2% of the aged care workforce (6). Their role focuses on assisting older people with the activities of daily living, such as toileting, showering, dressing, and eating. Recruiting and retaining PCWs in nursing homes has been a challenge in Australia for over a decade (9), and the majority are middle-aged women, many of whom are migrants (10). The most recent Australian Aged Care Workers Survey (6) found that many respondents reported their country of birth as Australia (56%), and 40% reported speaking a language other than English.^4^ In May 2023, the Commonwealth government introduced the Aged Care Industry Labour Agreement to assist with recruiting PCWs from overseas to address workforce shortages in the aged care sector (11). Currently, there are 11 Aged Care Industry agreements signed with Australian nursing home providers that cover 2,000 temporary visa places and 4,000 permanent places over five years (11). While nursing home providers indicate they wish to increase the use of migrant PCWs, identified barriers include issues related to visa sponsorship procedures, the inability to offer sufficiently attractive salaries, and the difficulty of attracting PCWs to work in regional, rural, and remote areas (12). Research has highlighted the challenges posed by the employment of migrant PCWs, including limited English language, which may affect their communication and interactions with colleagues and older people, particularly their ability to follow care instructions and report issues that impact the relational aspects of care, and older people’s safety (13–15).

There is evidence that suggests PCWs are unprepared educationally for the complex care needs of older people in nursing homes (16, 17). There is no formal industry standard for an entry-level education qualification to work as a PCW, nor any government-mandated specific qualification type or level. The 2024 Aged Care Workers Survey found that 30% of PCWs hold a Certificate III in Individual Support (Ageing), followed by 27.8% who hold a Certificate III in Aged Care (6).^5^ Both these certificates are basic vocational and educational training (VET) qualifications (18), where the curricula cover essential skills, such as providing individualised support, facilitating empowerment, supporting independence and well-being, and following safe work practices. The qualification is designed to prepare individuals to provide person-centred support to older people in various settings and requires the completion of 15 units of study and at least 120 h of work placement (19). However, the literature indicates that PCWs have little understanding of person-centred care (20, 21), which is considered the cornerstone of quality care; and they are not fully aware of the implications of missed care such as ADLs or other care tasks, and do not see it as their responsibility (3). As early as 2018, PCWs in nursing homes were reported as being incapable of meeting the care needs of older people (17, p.5) with knowledge and skill gaps identified in: basic care, such as hydration and nutrition; health knowledge key to the sector such as dementia and end of life; and interpersonal skills, such as communication (17, p.26). This report noted the pressing need for education and training to boost the competencies and skills of current PCWs through further education and by modernising and realigning vocational education and training of future PCWs (17, p.15–22).

The Australian aged care sector has faced intense scrutiny in recent years with reports of serious harm to older people (4, 22) despite earlier reviews of the sector workforce (17, 23). This scrutiny culminated in the Australian Royal Commission into Aged Care Quality and Safety (the Commission) in 2019 (4). The Commission received over 10,000 submissions listing examples of poor-quality routine and complex care from multiple stakeholders, with an estimate of a third of all older Australians living in nursing homes experiencing poor-quality care (4, p.72). The final report from the Commission noted that a highly skilled care workforce was vital to a safe, quality aged care system and made important workforce recommendations linking high-quality care to workforce planning and staff education and training. Recommendations for workforce education and training focused on ensuring PCWs were equipped with the skills and knowledge needed for current and future aged care needs, supporting their professionalism, improving career opportunities, and increasing wages and conditions (Recommendation 77) (4).

The World Health Organization states that person-centred, high-quality care of older people is facilitated when nursing home care staff work collaboratively, integrating care with a common purpose (24). Research indicates that an integrated approach to older people’s care that includes ongoing staff education (25) improves the quality of care and health outcomes and decreases unnecessary hospitalizations. This paper presents, as case studies, two education packages developed for PCWs (26), who are an integral part of the nursing home care team, to improve their knowledge and confidence in providing optimal care to older people.

### Overview of the pedagogy

Both education packages were developed for nursing home care staff, primarily PCWs who are adult learners with various educational backgrounds. When developing learning materials for PCWs in nursing homes, incorporating both andragogy and heutagogy can be highly effective. Andragogy, the method and practice of teaching adult learners, emphasises the importance of self-directed learning, practical relevance, and drawing on the learners’ experiences (27, 28). This approach ensures that education and training are directly applicable to the PCWs’ daily tasks and leverage their existing knowledge and skills. Heutagogy focuses on self-determined learning, encouraging learners to take control of their educational journey. This pedagogy promotes critical thinking, problem-solving, and adaptability, which are crucial for PCWs who must respond to the diverse and evolving needs of aged care residents. By using these two pedagogies, our education and training programs create a more engaging, relevant, and empowering learning experience for PCWs. Both education packages are focused on relevant, contemporary service delivery within the aged care sector.

The first education package - *Recognise & Report* - was created in response to increasing reports on the inadequacy of PCW education (3, 16, 7, 22). The *Recognise & Report* content was developed in 2017 after research that explored the educational needs of PCWs, as well as the acceptability and feasibility of delivering education to PCWs in nursing homes. A self-selected convenience sample of 32 care staff (see Table 1) from two geographically diverse nursing homes were interviewed. The participants were largely representative of the nursing home workforce (6, 10). The educational background of the PCWs varied across both nursing homes especially among overseas born PCWs at nursing home A (two overseas educated medical doctors who could not get registration in Australia, an engineer, and an accountant) compared to the PCWs at nursing home B who were mature aged women returning to the workforce, few with tertiary education or formal qualifications. All participating PCWs either had or were completing a Certificate III qualification (18); however, there was a great diversity in the length, content, and delivery of their courses. Despite the different educational backgrounds, analysis of the interviews identified barriers to further education for this workforce group, including the cost of education and the lack of time to attend (see Table 2), which have been cited before. The research also identified multiple areas for further education and skills and PCWs’ preference for learning in small interactive groups (see Table 2).

**Table 1:** Recognise & Report education development participants.

	Nursing Home A *n* = 14		Nursing Home B *n* = 18	
Geographic location	Metropolitan		Regional	
	***n***	**%**	***n***	**%**
Registered nurses (*n* = 7)*	4	*28*	3	17
Enrolled nurses (*n* = 5)	1	*7*	4	22
Personal care workers (*n* = 20)	9	*64*	11	61
Female gender	13	*93*	18	100
Australian born	5	*36*	18	100
Length of certificate III education	12 to 20 weeks		20 to 52 weeks	
Median length of service in nursing home sector	8 years Range (3 mths to 40 yrs)		7 years Range (2 mths to 20 yrs)	

*RN includes 3 clinical care coordinators and a manager.

**Table 2:** Perspectives on education package (Development research).

Expressions of support for PCW education	
Basic education poor	Their basic education is exceptionally poor. When they come into nursing homes, they do not have the knowledge to look after residents. It’s all paper-based knowledge. They are taught mostly generic knowledge about occupational health and safety and that you must be kind and respectful (RN/Education Officer, Service B).
PCWs feel and are restricted	I think they would love it. I think they do feel restricted in what they can do. If they report something, they hope it’s passed on or documented. Whereas, if they were able to document it themselves, they know they have put it in writing (RN, Service B).
Would improve PCW self-esteem, clinical knowledge, and accountability	I think it’s very important [for PCWs] to be able to have the knowledge and skills to recognize and then report. The RN or the EN is busy, they are giving medications and responding to doctors, so things can be missed. They cannot always get to see all the residents in one day. So, a few days might go by before they might assess a resident, you know, to see any changes in them. I think it’s [education] very important. It’s good for their self-esteem and their clinical knowledge, it gives them more accountability (RN/ Manager Service A).
Education for all enables everyone to benefit	The more information you can give to everybody, the better the residents will feel because they feel like they are in safe hands. Education is key, so the more education that everybody has, the better the job is (PCW, Service B).

Suggestions for PCW education content	
Assessments	As PCWs we do not get trained to do observations (vital signs), but I’d like to know what that number means on the blood pressure machine. What is the normal temperature range? I’d like to feel confident in knowing what it means (PCW, Service B).
Dementia	I think PCWs need to have more training in how to deal with dementia-specific residents and the care that they give (EN, Service B).
Body systems	It [education] should be more all-encompassing on the human body, so you’d be able to tell if their blood pressure was getting low or too high or if they are [residents] not drinking, dehydration. What’s happening to the body? What going on? Not just what they look like, so we understand what’s going on in the body (PCW, Service B).
Skin	What normal skin looks like, what a cancer looks like, what herpes would look like. Why the skin tear needs to be reported immediately, and you do not put oxide or sticky tape on it. We’re not asking them to diagnose but saying, “These are all abnormal.” (EN, Service B).
Pain	Pain, recognising pain. They’re the ones who are doing the ADLs behind closed doors. Unless the RN or EN happens to go in, you do not see a lot. So you are relying on feedback which often is not forthcoming. And it’s basically because they do not have the skills (RN Clinical Coordinator, Service A)
Communication	I think mostly about communication. For like different countries. Residents come from different backgrounds, so you need to know how to talk to them. I think it’s just more important (PCW, Service A).
Documentation	What we need is PCWs to know how to look after people with very, very complex needs and how to read care plans and make changes (EN, Service B)

Preferred education delivery	
Face-to-face	I do not like online learning. I prefer it in front of me with somebody [delivering] and practical elements as well. I enjoy that, and I think it’s a better way to learn (PCW, Service B).
Hands on	I think more hands-on because some people are more visual, and they see, “Okay, that’s exactly how I have to do it.” If you see one person doing it and then someone else doing it, then you copy each other (PCW, Service A).
Group learning	I think the hands-on and doing it together. Doing with them [PCW], showing them what a normal, I do not think you can beat that (RN, Service B).

Feasibility	
Barriers Travel, cost, family responsibilities, other employment	I think more people would be keen if you do it in the facility. If you told me I had to travel, I do not think I would go (PCW, Service A). Sometimes, those courses are like $600 for a couple of days. I do not get enough money to do that (PCW, Service B). Personal responsibilities. A lot of PCWs have children, so they must get home (RN/Manager, Service A).
Enablers Onsite education provided by RACS	I’d like to do more education. If it was here, it’d be so much easier because I work in another facility as well, and I cannot keep cancelling shifts (PCW, Service B).

The second education package, called Aged Care Education & Training (ACET), was developed for PCWs, as well as nurses and allied health professionals working in nursing homes (26), in direct response to interim findings and recommendations from the Royal Commission.

### Learning environment

Teaching Objectives: Using a body systems approach, each module in the *Recognise & Report* education supports PCWs to understand how the body systems work; the ageing process and what happens to the body; how to recognize a change in an older person’s health and wellbeing; and how to report these changes verbally and in writing. The high-level intended learning outcomes of each *ACET* module relate to developing knowledge, skills, and attitudes and raising awareness (26). Users should be able to: demonstrate best practice in the care area; develop confidence and capacity to make informed decisions to improve care; build and translate knowledge to care; ‘stop and think’ about performance and to identify areas for improvement; work through the process of decision making in a timely way; and identify and access additional resources to assist with professional development.

Curricular Strategy: The education content and delivery mode of the *Recognise & Report* package were developed acknowledging the socio-demographic profiles, prior learning, and the specific educational needs of PCWs, and was designed to address the barriers identified in the research. The second education package, *ACET*, was developed with Victorian State government funding during the COVID-19 pandemic as part of their response to the Commission’s recommendations. The brief was to develop, implement, and deliver an online education package (due to COVID-19 restrictions) to build capacity and capability in the nursing home workforce.

Curricula Audience: Both packages had no prerequisite educational requirements and were designed to be delivered to adult learners who speak English and work in nursing homes. The primary target of the *Recognise & Report* education was PCWs, intending to enable them to recognise changes in older people’s health and to empower them to report these changes to the appropriate health professional. The *ACET* package targeted PCWs as well as nurses and allied health care professionals working in nursing homes and focused on three key areas recognised as significant competency gaps of the nursing home workforce identified by the Commission: dementia care, recognising and providing a palliative response to care, and oral hygiene and links to health and well-being.

Curricula content development and delivery overview: The *Recognise & Report* education package was modeled on previous education developed by the team for RNs in response to an identified gap in nurse education and skills in Australia – Comprehensive Health Assessment (CHA) of the older person (29). The education package uses the train-the-trainer (TTT) model and is designed to be delivered by a nurse at the nursing home. The TTT model has been recognised as a successful approach for developing trainers who can then train other individuals in skills or aspects of their professions to broaden the number of people trained (30) and is a cost-effective approach for efficiently training large groups of individuals. The education consists of eight modules (ten hours delivery) (Communication, Wellbeing, Movement and Mobility, Skin, Breathing, Eating, Drinking and Elimination, Mental Awareness, and End of Life) developed from the Standardised Care Processes (SCPs) which are a synthesis of evidence-based guidelines related to recognised clinical risks for older people in nursing homes (31). The modules can be delivered individually or as a suite over two days. The package includes slides for the modules - with text, case studies/vignettes, video clips, and images; workbooks with activities that participants can work through during the education; a list of activities to help make each module interactive and enjoyable; summary sheets (a snapshot of each module) for each PCW; and a facilitator’s manual that outlines each module including time to deliver, equipment needed, learning outcomes, and an assessment of knowledge for each module (true/false questions). A key component of the education modules is ‘Mrs Brown’ who lives in a nursing home, has dementia and multiple health issues, and whose husband has recently died. Mrs. Brown is presented in the introductory module and is used as a case study for PCWs to apply new knowledge throughout the eight modules. The education package was piloted in three different nursing homes in workshops with PCWs before being available more generally to the nursing home sector. The education package was delivered by a RN involved in developing the package in two delivery modes: Workshop 1 delivered all the modules over two days, and Workshop 2 delivered the modules each day over a five-day week. After receiving the education, thirty-three PCWs provided feedback on the education content, delivery, and type (modular). The PCWs preferred the face-to-face format, the interactive activities, and the workbook, which they could take with them for reference. The preferred delivery was over two days.

The *ACET* education package was developed for PCWs, as well as nurses and allied health professionals working in nursing homes. *ACET’s* curriculum framework uses a heutagogical approach (26–28) where the focus is on the learner. This approach has five key principles that suit the online environment: collaborative, active learning activities; learner-directed open-ended questions; a flexible learning journey; a learning journal/portfolio; and learner-driven and paced. It ensures that content is accessible to all workforce groups and enables end users to choose what and when they wish to learn. The education content for the three key areas was developed by subject matter experts and assessed using the Flesch Kincaid readability index (32), as language and readability were critical to the development and success of the education package. *ACET* was developed with an awareness of the differing levels of educational preparation across the workforce to ensure the content was neither too complex nor too simple. This education enables learners to self-assess by including reflection opportunities such as ‘stop and think’ quizzes and other activities. New *ACET* modules are progressively being developed and uploaded into the *ACET* suite, 27 in total. Users include national and international participants, 60% of whom identify as PCWs.

### Results to date

The *Recognise & Report* education has been tested for user acceptability in the sector three times. Initially, during the development stage, and then in the refinement stage, it was delivered by an RN to 88 PCWs who work in supported residential services in Victoria, Australia. The feedback was positive. Participants reported that it increased or refreshed their knowledge and skills, and the most popular modules were mental awareness and end-of-life care. Participants reported that it increased or refreshed their knowledge and skills, and the most popular modules were mental awareness and end-of-life care. The final user acceptability testing (33) was undertaken in 2024. A telephone survey collected information about the useability and benefits of the education package for PCWs from the perspective of the RN’s delivering it. The education package was delivered in one metropolitan and 20 regional public sector nursing homes (*n* = 21) to 144 PCWs. In some nursing homes, the education was delivered multiple times. Twenty-three surveys were completed, representing 33 nursing homes (several of the nurses had delivered the education package in more than one nursing home). Content analysis of nurses’ experiences of delivering the education indicate the education met its target. RNs reported that the education was high quality, relevant, relatable, and easy to use, and the content was adaptable to meet the different learning styles of the PCWs and their time availability. They added that targeted education was needed by PCWs and that it helped the PCWs to identify and report health problems, improved their awareness of person-centred care, and validated their role and work (see Table 3).

**Table 3:** Nurses’ feedback on the Recognise & Report education.

Perceptions of education package	Exemplar quote
Education is engaging, relatable, and relevant to aged care (31%) A good refresher to improve resident care (39%) Able to be tailored, language PCW appropriate (46%) Training allows for group discussion – increases learning (31%)	A great refresher workshop. Language for documentation in case of deteriorating residents’ health, so they learnt important language and good documentation advice. Refreshing our knowledge to help care for my residents. Extremely informative and valuable. Valuable information regarding end-of-life care. They liked the case studies. Where to get help when the resident is in distress at end-of-life care. Engaging and insightful, it’s excellent to have this education (Education Coordinator & Clinical Support Nurse, Regional PSRACS with 22 PCWs).

Benefits RACS	Exemplar Quote
Provides free and appropriate education for PCWs (85% of responses) Education supports team building (53%) Supports person-centred care through communication, assessment, and reporting (46%) Convenient and cost saving (23%)	It was good, I think it was well set out. We tried once just running one of the modules on its own, and it was much better when we did it altogether and used the Mrs Brown case study. I think bringing the team together, obviously we were coming out of not much face-to-face learning over the last few years (COVID-19 pandemic) and this allowed us to bring staff together for two full days of education, so that was well received. There was no cost because it was all in-house. We did bring everyone together from PCW, EN, and RN level, so that was team building. It covers a lot of the national standards, so it does tick boxes where we need to have training in our nursing homes (Learning and Development Manager, Regional PSRACS with over 50 PCWs).

Benefits PCWs	Exemplar Quote
Education is needed for all new staff to the RACS (69% of responses) Re-enforces role definition (46% of responses) Education helped PCWS think effectively and to identify changes, and improves confidence in reporting (46%)	I received some emails from the PCWs about this package because they were appreciative of the way we delivered the package and even the content of the package. They were impressed. They mentioned it helped them to think more effectively, and when they identify the changes, they can relate it to different conditions now (Aged Care Practice Development Nurse, Regional PSRACs with over 20 PCWs).

Feedback from PCWs	
Positive overall (82%) Those who attended spoke highly of it (36%) PCWs appreciated having access to training and learning opportunities (64%) Great interactive education(19%)	The feedback that I’ve received has been “Oh I did not know that” or “Oh that’s a really good refresher.” It’s been great. After each week, we do it [education] on a Monday, we’ll talk about it, and I will ask them how did that go? How did they feel about that? Did they learn anything? Is there anything else that we can touch on? And the feedback’s always very, very positive. It’s simple enough, it does not sort of go over their heads. They’re all getting confident with that as the modules go along. They’ll say, “Actually that’s a really good way to remember when I’m doing my notes: what did I hear, what did I see, what did I feel?” (Clinical Support Nurse, Regional PSRACS with 7 PCWs).

What has changed in RACS	
PCW asking more questions (46%) Raised PCW awareness on how to alter their care practice (34%)	We’re seeing different questions being asked for our palliative residents. They were, “Well, that should not happen like that and how do I get this information?” So they are then asking for further skills to build their confidence to be able to take their data and their concerns to somebody else, which I think has been a really big positive for them as well (Clinical Nurse Educator, Regional PSRACS with 39 PCWs)

**Figure 1:**
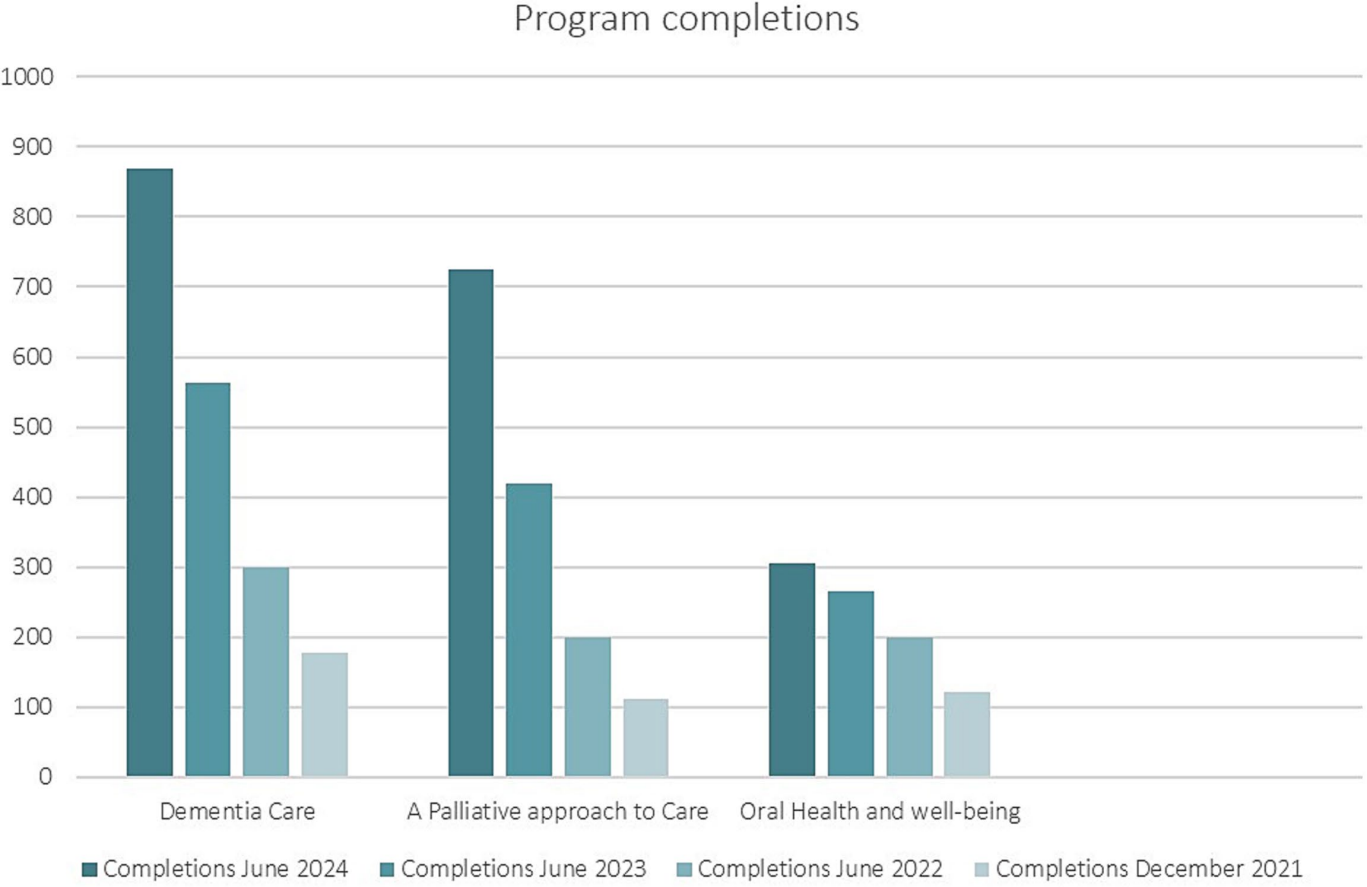
Completions of the three most popular topics 2021–2024.

The *ACET* education went live online in July 2021. Like the first education package, *ACET* underwent user acceptability testing during its design and development. In a drop-down feedback option, *ACET* users report the package to be easy to navigate and include the right amount of information in each module presented in a simple format. The content allows users to choose their learning journey based on self-assessed needs. Many nursing homes suggest modules for their staff based on the profile of the older people living in the nursing home. Some nursing homes have made completion of *ACET* modules mandatory education for their staff. Raising awareness of key clinical elements of clinical care within the sector is the aim of this package, and users report improved awareness and greater understanding of these issues. The inclusion of resources, such as the SCPs, and links for further information is welcomed by users wanting more information. Data collected from the *ACET* online platform includes registered user numbers (enrolments); module access; completion of modules/programs; place of employment and postcode; role (work role); number of years working in residential aged care; and geographic location or country of the user. Regular analysis of program usage shows that the original modules are the most popular (see Figure 1). Despite an abundance of dementia education available online, these modules remain the most popular within this education package. There have been 3,429 active users of *ACET* since it was launched in July 2021.

## Discussion

This manuscript describes two education packages developed to improve the knowledge and confidence of PCWs working in nursing homes. For more than two decades, nursing home workforce issues, particularly staffing levels and skills mix, have been raised nationally (3, 9, 16, 20, 34–38) and internationally (39, 40). The sector faces intense challenges in maintaining a skilled workforce for a population with complex care needs. PCWs comprise the largest aged care workforce group in the Organization for Economic Co-operation and Development (OECD) countries, typically high-income with advanced infrastructure (39). However, a recent global report observed that “*nursing home care workers do not always have enough training on geriatric conditions…and [in the of] management of emergencies,” which directly impacts the quality of care to older people* (40, p.14). The 2018 Aged Care Workforce Strategy Taskforce also identified significant gaps in the competencies of Australian PCWs and highlighted the need for the VET sector to produce a highly skilled and adaptable workforce (17).

The lack of appropriate education and training for PCWs worldwide was strikingly illustrated during the COVID-19 pandemic, which exacerbated pre-existing staffing challenges in the nursing home workforce and arguably led to increased rates of infection and even deaths among nursing home residents (41–43). While outbreaks of infections are not uncommon in Australian nursing homes, especially seasonal influenza and gastroenteritis, deficiencies in PCW education, especially understanding the need for and use of infection control measures, was one of many factors thought to have contributed to the disproportionate number of deaths in nursing homes compared to the general population - nursing home deaths accounted for about 75% of Australia’s total COVID-19 mortality (44). Similarly, in the United States (US), there is a lack of education and understanding about infection prevention and control among certified nursing assistants (CNAs)^6^ in nursing homes (45). In the first seven months of the COVID-19 pandemic in the US, non-adherence to personal protective equipment and mask use, appropriate transmission-based precautions, and hand hygiene by staff were the most common reasons for COVID-19 infections in nursing homes (46). In 2023, following the COVID-19 pandemic and the recommendations from the Commission, the most common work-related training completed by PCWs was infection prevention and control (6).

In Australia, where older people living in nursing homes have increasingly complex care needs (1), there remains a distinct disconnect between the current PCW education and training and the care needs of these older people. A review of current minimum educational requirements for PCWs may be warranted to ensure that they are educationally and linguistically prepared to provide the care older people in nursing homes require. The Commission recommended mandating a minimum Certificate III qualification for all PCWs, a recommendation the government has still not accepted despite the latest worker survey indicating up to 40% of PCWs do not hold this qualification, and 5% of PCWs do not hold any post-secondary school education qualification (6). The VET curricula should be improved and standardised to meet the education requirements of PCWs to detect and report deterioration in older people (47) and to have knowledge and skills in all aspects of resident care. This is especially relevant as part of the Australian Government’s response to recommendations from the Commission involves strengthening the current Australian Aged Care Quality Standards. Two of the new strengthened standards to be introduced in July 2025 indirectly refer to the need for further education of the nursing home workforce so all staff can detect and respond to deterioration. Standard 3 describes the required changes to assessment and care planning and focuses on multidisciplinary approaches to organising care (48). Standard 5 describes the responsibilities of providers to deliver safe and quality clinical care and focuses on nursing home staff understanding the importance of person-centred care to reduce and manage clinical risks (49). While strengthening the quality standards aims to address the quality of care provided in nursing homes, it does not address the barriers for the current PCW workforce to undertake further education. Both education packages presented cover the key clinical risks within the sector, which contribute to raising awareness of these issues among the workforce and addressing known barriers to education among this workforce group.

For the current nursing home workforce, continuing education would seem paramount. While continuing education has been a mandated requirement for registration for other healthcare professions in Australia since 2010 (50), it does not apply to PCWs because they are not registered and have no standardised scope of practice. In Australia, PCWs have consistently cited the lack of career progression and ongoing education and training as the main reasons for leaving the sector (51, 52). The barriers to ongoing education for PCWs are well researched (45, 51). These include: time – the demands of paid employment and family responsibilities; staff shortages – limitations around the number of staff who can attend education because of low staff numbers which means covering care is difficult when staff attend education; cost – both the individual and the nursing home may have financial constraints that limit access to education; inconvenient time and location making it difficult to attend; a lack of organisational support for further education; and a lack of interest or commitment among PCWs. Both the education packages presented are designed as ongoing education for PCWs already working in nursing homes. The packages were developed to accommodate the known barriers to ongoing education among PCWs, as well as their different education backgrounds and learning needs, and the modules are updated every two years to incorporate new evidence.

In line with findings from the original research and user acceptability testing of the *Recognise & Report* education package, others report that PCWs prefer face-to-face interactive learning (47, 53, 54). However, the COVID-19 pandemic has resulted in a surge in the uptake of online education (55). The use of online learning is now comparable to traditional forms of workplace learning, higher than trainer-led training (36%) and in-house education (51%) (56). Perhaps the high number of PCWs accessing the *ACET* modules reflects this trend.

Providing education in an accessible format for the aged care workforce is critical to uptake and completion. These two education packages provide PCWs with choice and flexibility: either modular learning delivered face-to-face in the context of care delivery or freely available online learning that can be undertaken in their own time. Both provide autonomy and empowerment. Education for PCWs must be designed with the learner in mind and include relevant strategies for diverse needs and educational backgrounds. The two packages focus on adult learning pedagogy that emphasises creating engaging, relevant learning experiences. Each package, delivered differently, caters to the unique needs of adult learners, promoting engagement, retention, and practical application of skills. The education modules in both packages, a synthesis of evidence-based guidelines related to recognised clinical risks for older people within the nursing home sector (31), should provide PCWs with the capacity and capability for increased recognition of deterioration and understanding of the individual care needs of older people.

The Australian Government needs to urgently mandate minimal educational requirements for PCWs to ensure they have the education and skills to provide the care older people require; require PCWs to be registered with a defined scope of practice; and make ongoing education for PCWs compulsory. These measures will align PCWs with the requirements of nurses and allied health professionals working in nursing homes, strengthen the nursing home workforce, ensure PCWs are better valued for the important work they do, and provide them with greater professional development opportunities and improved conditions (57).

### Limitations

Neither of these education packages presented have been formally evaluated, however, both included at least one user acceptability testing phase during their design and development. They remain popular in the sector, and the content of both education packages is updated every three years to reflect changes in evidence-based practice.

## Data Availability

The datasets presented in this article are not readily available due to ethics requirements, no data on the development of Education Package 1 is available for release. Requests to access the datasets should be directed to Jo Rayner, j.rayner@latrobe.edu.au.

## Footnotes

^1^Called long-term care homes and residential aged care services elsewhere.

^2^Aged care facilities in small rural towns and remote communities with fewer than 31 places may apply for an exemption from the responsibility until 1 July 2024 if they have appropriate alternative clinical care arrangements in place.

^3^The equivalent of Certified Nursing Assistants in the United States.

^4^While this was a self-report survey, the response rates for this report were very low and many questions were unanswered, so results need to be viewed cautiously as early reports suggest this number might be higher.

^5^As above. The 2020 and 2016 Aged Care Workforce Censuses reported higher proportions, but care needs to be taken with comparisons as the surveys were collected on different bases. 2016 was self-report and 2020 was a report about the workforce by nursing homes.

^6^The equivalent to PCWs.
