# Supplementary material for: Models of education for care workers in Australian nursing homes: improving the care of older people
Source: Front Public Health. 2025 May 19;13:1584889. doi: 10.3389/fpubh.2025.1584889 (PMC12127136; doi:10.3389/fpubh.2025.1584889)
Supplement: Supplementary file 1 [file Supplementary_file_1.docx]

| **Appendix A: Interview Guide PCWs** | |
| --- | --- |
| **Questions** | **Prompts** |
| Can you tell me what you do in this facility? | Role |
| How long have you been working here? | First RACF  Previous work history |
| How many days/shifts do you work? | Hours  Days  Casual/part-time |
| Tell me about a typical shift. | Work duties - routine, reporting and recording on charts, admission assessments? |
| How do you know what your role and duties are? | Induction  Worked in the sector before |
| What education or training did you have? | Cert 3  Cert 4  Other  Specific aged care education/training |
| Do you think your education/training prepared you to recognise any changes in a resident’s health? |  |
| Do you have opportunities for ongoing/further education? | Onsite  Funded  During worktime |
| How confident do you feel about recognising changes in residents’ health? |  |
| Can you tell me about a time when you recognised changes in the health of a resident you were looking after? | Delegate  Responsibility  Report |
| Did not feel you had the skills or confidence to do so recognise and report this change? | If yes, explain  If no, why |
| How important do you think it is to know the resident you are looking after to be able to recognise changes in their health? | Why |
| How did you recognise change? | See, Hear, Smell, Touch, Other |
| What do you do when you find changes? | To whom report  Record |
| What would help you to do this better? | Initial education  Ongoing education |
| How do you prefer to learn new skills or knowledge? | Visual/Written  Online/Face-to-face  Group/individual |
| Tell me what topics/issues you think should be included in ongoing education for PCWs. |  |
