# Supplementary material for: Models of education for care workers in Australian nursing homes: improving the care of older people
Source: Front Public Health. 2025 May 19;13:1584889. doi: 10.3389/fpubh.2025.1584889 (PMC12127136; doi:10.3389/fpubh.2025.1584889)
Supplement: Supplementary file 2 [file Supplementary_file_2.docx]

**Appendix B: Evaluation of Recognise and Report Education Package**

This evaluation aims to measure the residential aged care service (RACS) utilisation of the education package, delivery by nursing staff, and benefits to RACS.

**Section 1: Demographics of the facility**

1. Number of beds: …………….
2. How many RNs and ENs are employed at this facility: ………..
3. How many Personal Care Workers (PCW) are employed: ..........
4. Geographical location type: Rural / Regional / Metro

**Details of the facilitator**

1. Position title of the nurse delivering the education package ……………………………….
2. Years’ experience working as a nurse: ……………………
3. Years worked specifically in the aged care sector: …………………………
4. Formal qualifications/professional development: ..........................................
5. Formal education/training facilitating groups of people: Yes / No
   1. If yes, please provide further details………………………………………………………………..
6. Previous experience sector facilitating training workshops: Yes/No
   1. If yes, please provide further details………………………………………………………………..

**Section 2: Delivery of the education package**

Please select which of the following reflects the number of modules delivered since receiving the package:

1. None
   1. If none, why? **Evaluation completed**.
2. Some (at least 1 but less than 8).
3. All (Modules 1 to 8).

If some or all, select which modules have been delivered so far:

1. **Module 1: Communication** YES/NO

If yes:

- 1. How many times has this module been delivered?
  2. How many PCWs have completed this module?

1. **Module 2: Wellbeing** YES/NO

If yes:

- 1. How many times has this module been delivered?
  2. How many PCWs have completed this module?

1. **Module 3 Movement & Mobility** YES/NO

If yes:

1. How many times has this module been delivered?
2. How many PCWs have completed this module?
3. **Module 4: Skin** YES/NO

If yes:

- 1. How many times has this module been delivered?
  2. How many PCWs have completed this module?

1. **Module 5: Breathing** YES/NO

If yes:

- 1. How many times has this module been delivered?
  2. How many PCWs have completed this module?

1. **Module 6: Eating, Drinking & Elimination** YES/NO

If yes:

1. How many times has this module been delivered?
2. How many PCWs have completed this module?
3. **Module 7: Mental Awareness** YES/NO

If yes:

1. How many times has this module been delivered?
2. How many PCWs have completed this module?
3. **Module 8: End of Life** YES/NO

If yes:

1. How many times has this module been delivered?
2. How many PCWs have completed this module?

**Details of delivery of education package**

1. The 8 modules of were delivered in the order (Modules 1-8): YES/NO

If yes: **Go to Section 3**

If no: Go to next question

1. Which of the following statements best reflects how the education package was delivered (more than one can be selected):
2. The facilitator delivered individual module(s) (not in any order) based on their choice
3. The facilitator delivered individual module(s) (not in any order) based on PCWs choice
4. The facilitator has delivered individual module(s) (not in any order) based on the RACS manager's choice

**Section 3: Resources used/not used**

1. What resources included in the education package were used during the workshops:
2. Power-point slides YES/NO
   - If no, why not?
3. Knowledge questions and answers for each module YES/NO
   - If no, why not?
4. True / False games YES/NO
   - If no, this not?
5. Flash cards YES/NO
   - If no, why not
6. Participant booklets (1 per participant) YES/NO
   - If no, why not?
7. Summary Sheets for each module YES/NO
   - If no, why not?
8. USB YES/NO
   - If no, why not?
9. CD YES/NO
   - If no, why not?
10. Microsite YES/NO
    - If no, why not?

**Section 4: Experiences of using and perceived benefits of the package**

1. Tell us about your experience of using the education package.
2. Tell us about any suggestions for improving the facilitator manual that would improve the delivery of this package (more effective or easy).
3. Tell us if there are any parts of the facilitator manual that you found particularly useful.
4. How, if at all, has the education package benefited the RACS?
5. How, if at all, has the education package benefited the PCWs who have completed some or all of it?
